# Reliable, efficient, and scalable photonic inverse design empowered by physics-inspired deep learning

**DOI:** 10.1515/nanoph-2024-0504

**Published:** 2025-01-27

**Authors:** Guocheng Shao, Tiankuang Zhou, Tao Yan, Yanchen Guo, Yun Zhao, Ruqi Huang, Lu Fang

**Affiliations:** Shenzhen International Graduate School, 12442 Tsinghua University, Shenzhen 518071, China; Department of Automation, Tsinghua University, Beijing 100084, China; Department of Electronic Engineering, Tsinghua University, Beijing 100084, China; Beijing National Research Center for Information Science and Technology, Tsinghua University, Beijing 100084, China; Institute for Brain and Cognitive Sciences, Tsinghua University, Beijing 100084, China

**Keywords:** inverse design, physics-inspired deep learning, optical computing, optical neural networks

## Abstract

On-chip computing metasystems composed of multilayer metamaterials have the potential to become the next-generation computing hardware endowed with light-speed processing ability and low power consumption but are hindered by current design paradigms. To date, neither numerical nor analytical methods can balance efficiency and accuracy of the design process. To address the issue, a physics-inspired deep learning architecture termed electromagnetic neural network (EMNN) is proposed to enable an efficient, reliable, and flexible paradigm of inverse design. EMNN consists of two parts: EMNN Netlet serves as a local electromagnetic field solver; Huygens–Fresnel Stitch is used for concatenating local predictions. It can make direct, rapid, and accurate predictions of full-wave field based on input fields of arbitrary variations and structures of nonfixed size. With the aid of EMNN, we design computing metasystems that can perform handwritten digit recognition and speech command recognition. EMNN increases the design speed by 17,000 times than that of the analytical model and reduces the modeling error by two orders of magnitude compared to the numerical model. By integrating deep learning techniques with fundamental physical principle, EMNN manifests great interpretability and generalization ability beyond conventional networks. Additionally, it innovates a design paradigm that guarantees both high efficiency and high fidelity. Furthermore, the flexible paradigm can be applicable to the unprecedentedly challenging design of large-scale, high-degree-of-freedom, and functionally complex devices embodied by on-chip optical diffractive networks, so as to further promote the development of computing metasystems.

## 1 Introduction

Optical computing hardware [1], [2], [3], [4], [5], [6], [7], [8], [9], [10], [11], [12], [13], [14], [15], [16], [17], thanks to the advantages of low latency, minimal power consumption, high parallelism, and high speed over electronic implementations as well as the elimination of digital-to-analog conversion as a form of analog computing, might boost the development of artificial intelligence in the future. At present, optical neural network (ONN) is prevalently implemented through interference based on MZIs [10], [11], diffraction using trained modulation units [1], [2], [3], [4], [5], [6], [7], [8], [9], wavelength division multiplexing enabled by micro rings [12], [13], [14], [15], [17], etc. However, owing to their large footprint, the integration density and scale of these optical neural networks is remarkably restricted.

Artificially designed subwavelength structures, known as metamaterials [18], [19], [20], [21], [22], [23], [24], [25], [26], may dramatically increase the integration level because of its subwavelength size of meta-atom. Interaction between light and metamaterials allows excellent phase and amplitude modulation of the optical wavefront, which has wide applications in the fields of focusing [21], imaging [22], [23], vortex generation [24], [25], spectroscopy [26], etc. It has a more compact size than conventional spatial light diffractive slabs, with highly integrated capabilities and multiple layers of metamaterials cascaded to realize on-chip all-optical diffractive neural networks. Diffractive optical computing metasystem [4], [5], [6] utilizes multilayer metamaterial structures to flexibly modulate the wavefront and might become the next-generation computing hardware platforms by virtue of its compact size, high computational density, and low power consumption.

**Figure 1:**
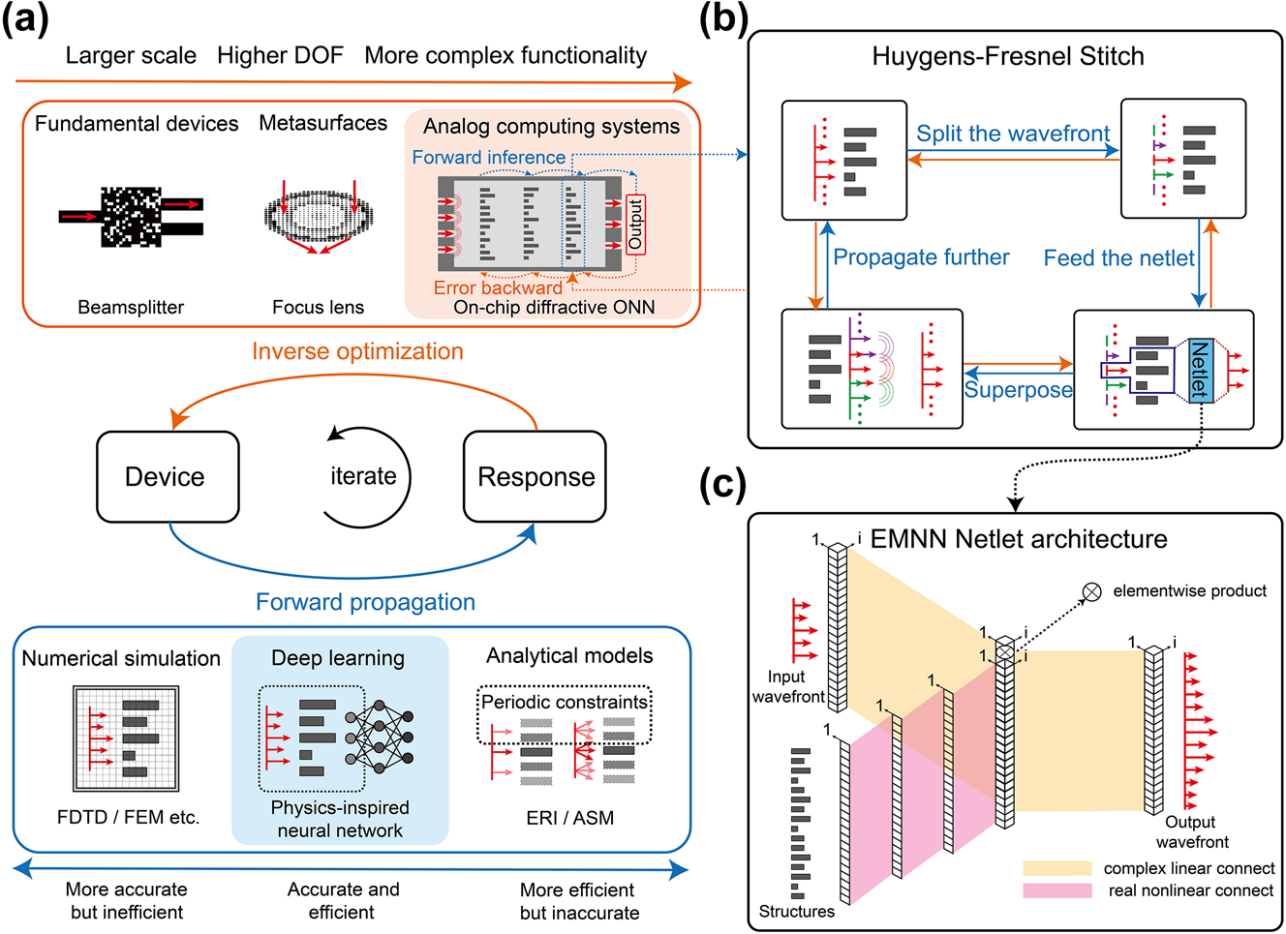
The conceptional diagram of inverse design empowered by physics-inspired neural network. (a) The schematic illustration of inverse design algorithms. Devices are optimized to produce desirable EM responses through iterative inferences and adjustments. Large-scale, high-design DOF, and complex functionality of the device along with challenging forward modeling are the major obstacles faced by inverse design. The proposed method aims to settle these issues. The core of the methods is Huygens–Fresnel Stitch (b) and EMNN Netlet architecture (c). (b) Huygens–Fresnel Stitch, a strategy to scale the forward model to arbitrarily large size. Enlightened by the Huygens–Fresnel principle, it manages to physically concatenate local wavefronts to form one large global field, enabling the method to be flexibly applicable to the device of nonfixed size. (c) EMNN Netlet architecture, which produces the output wavefront based on the input wavefront and structures. It takes the variable input field into account in an inverse design network for the first time. With physics-inspired design, it succeeds in giving full-wave prediction both accurately and efficiently and manifests great interpretability and generalization ability.

Earlier designs of metasurface, which are either based on intuition by manually adjusting parameters or the prior knowledge by employing an analytical model [27], [28], takes large amounts of trial and error, making the design process inefficient and arduous. Recently, inverse design provides a novel paradigm for device designs, which optimizes the design objective by adjusting the structure parameters so as to yield the desired optical responses, greatly improving the efficiency of the design and performance of the device [29], [30], [31], [32], [33], [34], [35], [36], [37], [38], [39], [40], [41], [42], [43], [44], [45], [46], [47]. Optimization algorithms mainly include heuristic algorithms [30], [31], [32], gradient optimization [33], [34], [35], [36], etc. Generally, these methods use iterative simulation numerical algorithms, such as finite element method (FEM), finite difference time domain (FDTD), and rigorous coupled wave analysis (RCWA), to obtain the forward evaluation of the device. These methods replace gradients with differences to solve the electromagnetic (EM) field constraints, Maxwell’s equations, and come with accurate and reliable results, but are computationally expensive in terms of both memory usage and computation time. The inefficiency of numerical methods can lead to time-consuming and costly inverse design where forward evaluations are constantly repeated in stepwise iterations. To mitigate the computational burdens, neural networks are utilized as a substitute for numerical simulation methods for forward evaluation to accelerate inverse design. In recent years, various deep learning models [37], [38], [39], [40], [41], [42], [43], [44], [45], [46], [47] are consolidated with inverse design in order to achieve better performance and higher efficiency, including discriminative architectures such as fully connected neural networks (FCNNs) [37], [38], [39], [40], convolutional neural networks (CNNs) [41], and recurrent neural networks (RNNs) [42] and generative models like variational autoencoder (VAE) [43], [44] and generative adversarial nets (GANs) [45], [46], [47]. These models, with complicated architectures and vast amounts of connections and weights, exhibit powerful learning capability, but meanwhile consume tremendous computing resources. In essence, these approaches are not initially devised to specialize in tackling how EM waves interact with metamaterial, thus leading to model redundancy and waste of computing resources. Apart from this, previous designs using deep learning have some other specific limitations. These works only focus on indirect properties like spectrum or transmittance rather than the optical field itself. Additionally, these methods show little flexibility because they disregard the variable input field and restrict the device size to be fixed. Moreover, in terms of the large-scale designs, the design space has an extremely high degree of freedom (DOF), which necessitates an extremely significant amount of simulation and optimization. As devices become larger in size and more complex in function, all these issues pose considerable challenges to inverse design (see Figure 1a).

Unlike the design of metalenses or some single-layer metasurfaces, the cascading of multilayer structures leads to the accumulation of physical modeling errors, and the effectiveness of analog computing systems is greatly affected. Hardware computational error arises from multiple reasons, mainly including modeling errors and fabrication errors. A significant modeling error inevitably leads to a larger hardware computational error. The accuracy of state-of-art analytical models fails to meet the requirements of large-scale computing metasystems. Thus, devices designed using analytical methods are significantly degraded when deployed in actual physical experiments, and this entails extra and complicated calibration to improve the performance. Lack of reliable and efficient physical modeling tools impedes the development of large-scale optical computing for performing complex tasks. To the best of our knowledge, there are currently no tools or models that can cope with such problems both reliably and efficiently.

Here, we propose and demonstrate a physics-inspired deep learning architecture termed EMNN (Electromagnetic Neural Network) to realize reliable, efficient, and flexible inverse design of large-scale, high-DOF, and functionally complex devices (see Figure 1b and c). EMNN is a method based on the principles of PINN (physics-informed neural network) [48], [49], [50], [51] concept, aimed at integrating data and physical models to understand high-dimensional spaces constrained by physical partial differential equations. Physics-inspired design is incorporated to the architecture so as to obtain strong physical interpretability and generalization ability. Distinct from conventional practices, our model gives direct, rapid, and accurate prediction of full-wave EM field distributions even with nonfixed device size or variable input fields given. Based on this method, computing metasystems (a linear system and a nonlinear system) are reliably and efficiently inverse designed to perform handwritten digit recognition and speech command recognition, respectively, verified by FDTD, proved to show high efficiency and high fidelity and surpass the comprehensive performance of state-of-art numerical, analytical, or other deep-learning-based methods. This work paves the way for the development of large-scale, highly integrated, and powerful optical intelligent computing.

## 2 Results

### 2.1 On-chip diffractive model

Light propagates in a fundamental slab mode (TM in this work), and the on-chip propagation process is modeled by two parts: the slab-mode propagation model and the diffractive modulation model. The slab-mode propagation operator *P*_*d*_ is derived from the angular spectrum approach and can be formulated as:

(1) $$u\left(z,d\right)=P_{d}\left\{u\left(z,0\right)\right\}=F^{-1}\left\{F\left\{u\left(z,0\right)\right\}\cdot H\left(f_{z},d\right)\right\}$$

where $u\left(z,d\right)$ represents the optical field at the position of $\left(x=d,y=t/2,z\right)$ , *F* and *F* ^−1^ are Fourier transform and the inverse Fourier transform, and $H\left(f_{z},d\right)$ is the transfer function:

(2) $$H\left(f_{z},d\right)=\left\{\begin{array}{c} \exp\left\{ikd\sqrt{1-{\left(\lambda f_{z}\right)}^{2}-{\left(\lambda f_{y}\right)}^{2}}\right\},\;f_{z}^{2}+f_{y}^{2}<\frac{1}{\lambda^{2}}\\ 0,\;f_{z}^{2}+f_{y}^{2}\geq \frac{1}{\lambda^{2}} \end{array}\right.$$

where *λ* is the wavelength, $k=\frac{2\pi }{\lambda }$ . *f*_*z*_ and *f*_*y*_ are the spatial frequencies. *t* is the thickness of the slab waveguide and *i* is the imaginary unit. In particular, the slab-mode propagation model has been discussed and well settled in many previous works [28], and its computing results are in near-perfect agreement with those of the FDTD solver.

Previously, physical modeling errors of on-chip optical networks are mostly attributed to the diffractive modulation model. Currently, the diffraction modulation can be modeled by analytical models or numerical simulation. Analytical models are derived from simplifications of physical process, such as the effective refractive index (ERI) method and the angular spectrum method (ASM). As much as they can rapidly compute the output field distribution, they fail to guarantee the modeling accuracy. While numerical simulation guarantees the precise modeling of the designed device, the device optimization time and computational cost would be astronomically high for large-scale, multi-layer cascaded, and high-DOF computing metasystems. In order to reach a balance between accuracy and efficiency, a physics-inspired deep learning model, EMNN, is proposed (see Figure 1a).

EMNN is composed of the local method Netlet and the global stitching strategy Huygens–Fresnel Stitch, (1) EMNN Netlet, a neural network with physics-inspired architecture, to predict the output wavefront based on given input wavefront and structure parameters. Netlet is the critical building block of the model’s effective prediction (see Figure 1c). (2) Huygens–Fresnel Stitch, a physics-inspired strategy that seamlessly stitches together the local responses predicted by Netlets to form a global response, empowering the network to handle arbitrarily large inverse design problems without increasing the complexity or degrading the performance of the design (see Figure 1b).

### 2.2 EMNN Netlet

**Figure 2:**
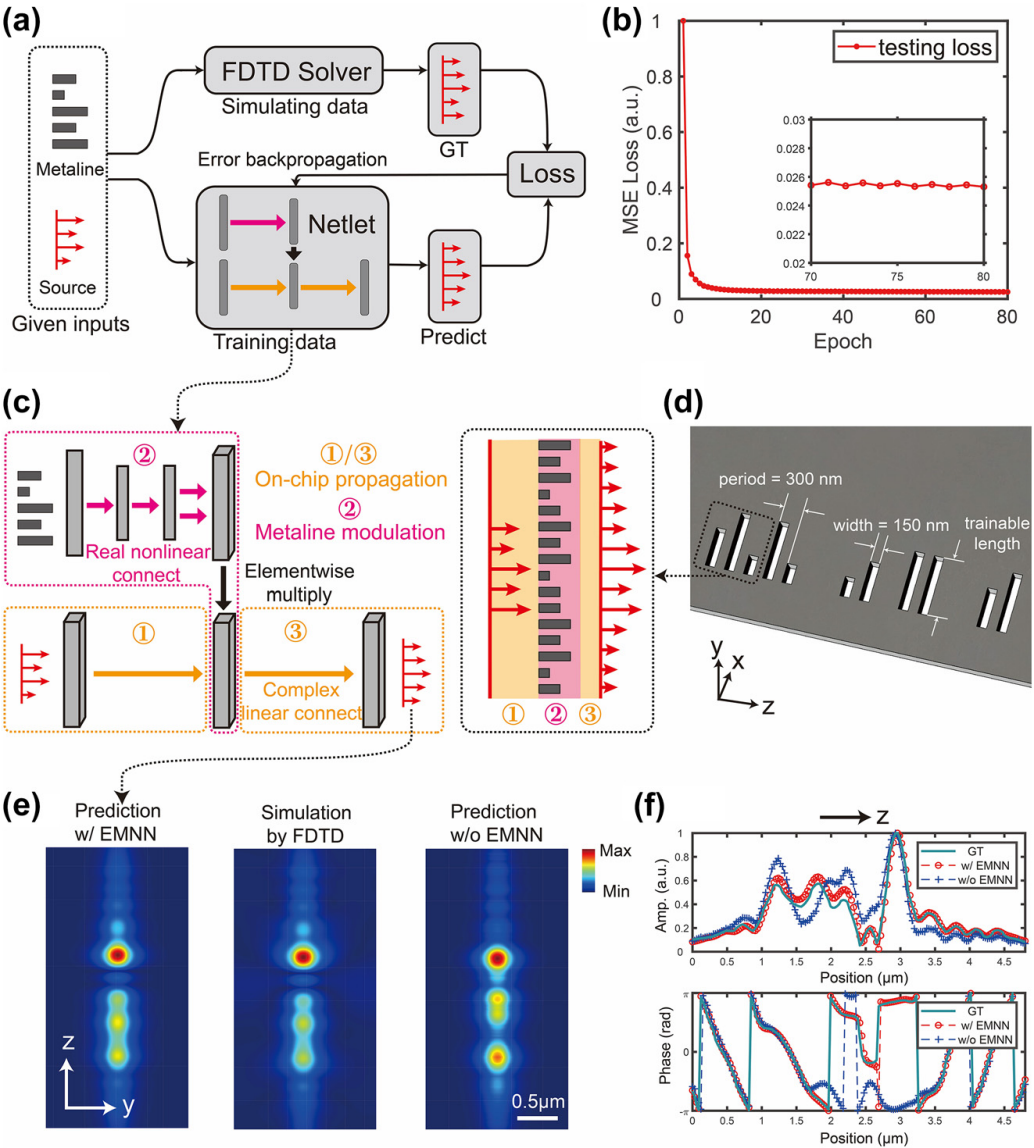
Training (a–b) EMNN, which exhibits strong physical interpretability (c–d) and generalization ability (e–f). (a) The schematic of the training procedure. Given the input field and structure, the FDTD solver and neural network are used for simulation and prediction, respectively. The simulation results obtained from the FDTD solver are considered as ground truth (GT) and used to compute the training loss, enabling gradient backpropagation for training EMNN. (b) The learning curve of the training. The testing loss converges after over 70 epochs of training. (c) The explainable correspondence between Netlet architecture and the light propagation process. Complex-valued linear connect of Netlet corresponds to the on-chip propagation of the light, while real-valued nonlinear connect can be associated with the modulation of the metaline. (d) The schematic view of one-layer metaline composed of meta-atoms. The thickness of the silicon membrane is 220 nm, and the width of the meta-atom is 150 nm. The period of the diffractive metaline is 300 nm. The amplitude and phase modulations of optical fields are controlled by varying the slot length. (e) The wavefront profile (in the *y*–*z* plane) generated by EMNN, FDTD, and a nonphysics-inspired network with a comparable number of trainable parameters and network layers. (f) The amplitude and phase comparisons of the wavefront generated by EMNN, FDTD, and the nonphysics-inspired network.

The architecture of this network is sketched in Figure 1c. A single Netlet is capable of tackling the wavefront modulation problem in a finite region of 900-nm width. Input–output pairs are randomly parameterized and then generated using FDTD simulation (see Figure 2a). A sum of 90,000 input–output pairs constitutes the training and test sets of the network, of which 80,000 serve as the training set, and 10,000 as the test set. It is worth noting that the simulation of the dataset for this network is quite sizable and requires a lot of computational resources. However, compared to those that need to repeatedly access the computationally costly and time-consuming FDTD solver, the simulation efforts in our method can be viewed as a one-kick investment. In other words, our network can be a quick-response substitute for inefficient numerical simulation.

Distinct from the previous practice of neglecting the input waveform, our network takes into account the variation of the input wavefront. Based on a dual-input architecture design, the network is able to predict the outcome of the output wavefront based on both the input wavefront and the structure parameter (see Figure 2a). The learning curve of training EMNN is shown in Figure 2b, indicating that the testing loss converges after around 70 epochs. In addition to that, it is worth noting that the input wavefront and the diffractive modulation are encoded in the complex-valued form instead of separating the real and imaginary parts. Following the constraints of physics, different parts of the network are connected in different ways. The input wavefront and the output wavefront are linearly connected using complex-valued weights while the input structure is nonlinearly connected using real-valued weights.

Generally speaking, neural networks are deemed as black boxes that lack interpretability. Instead, this physics-inspired neural network is elaborated to be physically interpretable, and there is a compelling explanation for physical meanings contained in this network architecture. The physical process of the wavefront modulation can be divided into three stages: (1) & (3) slab-mode propagation off the metaline (the distance is short but exists); (2) diffractive modulation process through the metaline (see Figure 2c). In the corresponding architecture of EMNN Netlet, we employ complex-valued linear connections to function as the slab-mode propagation process (1) & (3) and real-valued nonlinear connections to act as the diffractive modulation process (2).

Thanks to the physics-inspired design, the network exhibits great generalization ability. Under the condition of the same network depth, comparable numbers of parameters, and identical training set, EMNN Netlet possesses superior learning capability compared to a nonphysics-inspired network. Wavefront profiles computed by EMNN, FDTD, and the nonphysics-inspired method are shown in Figure 2e for the same test sample, which is generated in a different way from any sample in the training set (see more comparative simulations in Figures S9 and 10). EMNN successfully predicts results consistent with those simulated by FDTD, whereas the nonphysics-inspired method fails to achieve the same level of accuracy. Figure 2f indicates that the physics-informed EMNN manifests greater predictive accuracy in both amplitude and phase. Furthermore, EMNN Netlet inherently satisfies the linear response constraint of the physical system (see Figure S4). We also demonstrate the superior performance of EMNN over that of 2D-FDTD (see more discussion and the simulation setup in Figure S8 and Table S3).

### 2.3 Huygens–Fresnel Stitch

EMNN Netlet is capable of addressing diffractive propagation modulation for wavefronts within a width of 0.9 μm. However, the practical wavefront width of on-chip optical chips is significantly larger than 0.9 μm. For example, in the subsequent tasks we are undertaking, the design width is 300 μm, which is three orders of magnitude larger than the wavefront width that a single EMNN Netlet can address.

If a single neural network is directly applied to the 300-μm-long device, a series of problems will arise. First, the complexity of the problem soars. As the applied region grows linearly, the cost of training the network, including generating the dataset, undergoes a multiplicative increase. In other words, the computational cost required to train a single forward prediction network for a 300-μm-wide device is at least five orders of magnitude larger than that for a 0.9-μm-wide device. Furthermore, even if we manage to contribute a lot of effort to training this individual neural network, the network is not flexible and generic. Once the size of the device is changed, we will have to start training a new network again from scratch.

Inspired by the principles of optical diffraction propagation, the Huygens–Fresnel Stitch method is proposed to seamlessly stitch EMNN Netlets together. According to the Huygens–Fresnel law, during the propagation of an optical field, each point on the wavefront can be considered as a center source generating secondary waves. Subsequently, these secondary waves superpose coherently to form a new wavefront. Taking inspiration from this, we designed the following process for the Huygens–Fresnel Stitch method (see Figure 1b).

Divide a wavefront with a width of 300 μm into several segments of 0.9 μm width each (if necessary, zero-padding can be added on both sides of the wavefront). Each of these segments can be regarded as a separate point on the wavefront generating secondary waves. Input each 0.9 μm segment of the wavefront along with its corresponding structure parameters of width 4.8 μm into EMNN Netlet. This yields an output wavefront of width 4.8 μm. These wavefronts represent the envelope surfaces generated by the secondary wave sources. Coherently superpose the predicted output wavefronts to obtain the final wavefront modulation result of this 300-μm-wide diffractive metasurface for the input field.

The physics-inspired strategy allows our model to be applicable to design problems of arbitrary device size, without increasing the complexity of the problem. More importantly, this strategy aligns the process of stitching wavefronts with the real physical process, significantly reducing stitching errors compared with previous nonphysics-inspired counterparts (see Figure S6).

### 2.4 Handwritten digit classification with linear ONN

**Figure 3:**
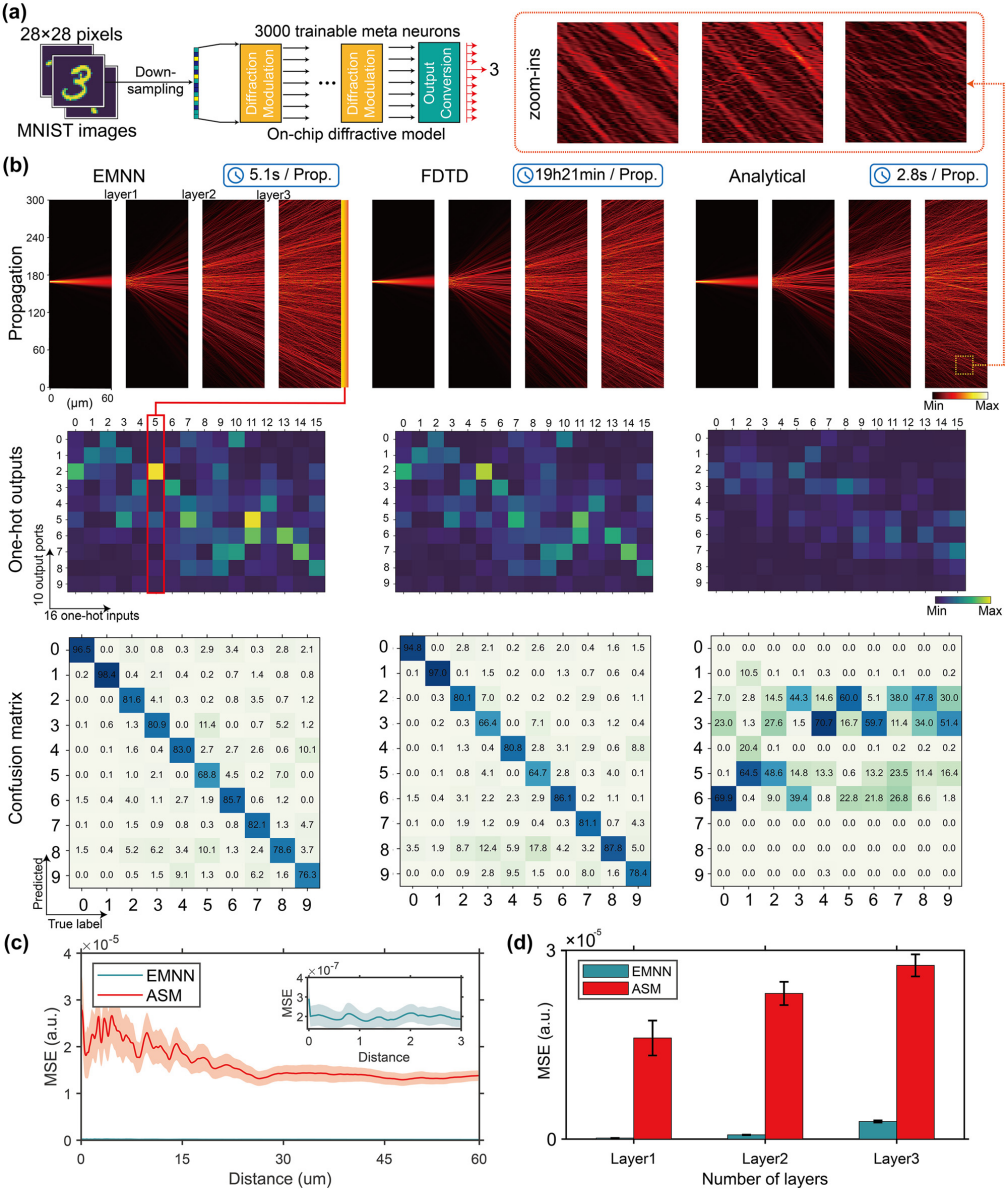
Three-layer ONN design on the task of handwritten digit classification. (a) The schematic of ONN architecture composed of preprocessing, diffraction modulation, and output conversion. (b) Comparison results using different forward inference methods (EMNN, FDTD, and ASM) on the same physical structure. Here, the propagation and outputs of FDTD serves as the ground truth. The first row shows the forward propagation profile with the sixth one-hot component inputted. The second row shows the output energy matrices from 10 output ports with 16 one-hot components inputted. The last row shows the confusion matrices. (c) Modeling error comparison versus propagation distance using EMNN and the analytical model, respectively. The modeling error of EMNN is two orders smaller than that of the analytical model. (d) Modeling error comparison versus the number of layers using the analytical model and EMNN. The modeling error of EMNN remains minuscule in spite of increasing layers.

On-chip diffractive metasystems are inverse designed to perform handwritten digit recognition tasks. The MNIST dataset is divided into a training set with 50,000 samples and a test set with 10,000 samples. Prior to entering the optical network, the data undergo some preprocessing steps. The (28, 28) grayscale images are first down-sampled to (4, 4) and then flattened into a (16) vector (see Figure 3a).

The on-chip optical neural network for completing this task comprises three layers of diffractive metasurfaces, as illustrated in Figure 3a. Each layer is 300 μm wide and has 1,000 independently controllable optical network neurons. The distances between the input surface and each layer as well as between the layers and the output layer are fixed at 60 μm. The handwritten digit signals are encoded into the amplitude of the input optical field. They are inputted into the planar waveguide through 16 inverse taper waveguides. The optical field propagates through the waveguide and then sequentially passes through the modulation of the three diffractive metasurface layers. Finally, the optical field reaches the output surface, where there are 10 output inverse taper waveguides. Ten intensity values are recorded through the output ports, and the classification result is determined by the highest intensity value of all 10 output ports.

EMNN is used as the forward propagation model to process the input signal. The ONN model is an arrangement of diffractive modulation models and slab-mode propagation models mentioned above. The structure parameters of the optical network are fed to the diffractive modulation models, and the actual optical response of the network is calculated through propagation. The calculated response is then compared with the target optical response using mean squared error (MSE), which serves as the loss function of the optical network. Besides, the loss function can also be considered as the design objective function for the inverse design task. The optimization is performed by backpropagating gradients to adjust the structure parameters of the optimized metasurfaces. In this process, the network is deployed on TensorFlow, and the Adam Optimizer [52] is chosen as the optimizer for the task.

Apart from the implementations of the EMNN method, the forward prediction model in the diffractive modulation model is replaced with the analytical method and FDTD simulation, respectively, to compare their predicting capabilities. ASM is considered the analytical model with best performance to date and the result calculated by ASM can be considered as the benchmark, while FDTD is a very reliable full-wave numerical method for EM field simulation and the simulated result can be considered as the ground truth.

Since the input signal is encoded in the field amplitude through 16 input ports, we can set the amplitude of one port to be 1 and the rest to be 0, thus providing 16 one-hot input signals. The top row in Figure 3b displays the propagated optical field when the 6th one-hot signal is used as the input. Zoom-ins are taken out for comparisons from the same areas in the final layer of propagation. As revealed in these figures (see Figure 3b), it can be seen that the propagation result predicted by EMNN is much closer to the results of FDTD simulation than that by ASM.

The second row in Figure 3b illustrates the intensity values from 10 output ports produced by the 16 one-hot inputs. These intensity values form a 10 × 16 energy matrix. The third row in Figure 3b illustrates the energy matrix obtained by subtracting the ground truth energy matrix (obtained from FDTD simulation) from each predicted energy matrix. A smaller value in this matrix indicates a stronger capability of addressing the forward problem.

The last row in Figure 3b shows the testing results of the optical neural network metasystem designed through inverse design on a handwritten digit test set. It can be observed that there is little evidence of performance degradation when substituting the EMNN method for FDTD solver. It is found that the simulated results align very well with the actual computational results. Conversely, if the forward solver is replaced with the analytical method, the result will deviate from the simulated result.

### 2.5 Speech command recognition with nonlinear ONN

ONN with simplistic architectures can only cope with relatively basic tasks. Only through the scaling-up and increasing complexity of optical networks [53] can they be capable of performing more intelligent optical computing tasks. The typical approaches involve increasing the number of neurons, the number of layers, and introducing nonlinear activation. However, since optical intelligent computing is fundamentally a realization of analog computation, the analog errors in optical computations pose the greatest obstacle to scaling up optical networks and achieving more sophisticated functionalities. Nevertheless, by introducing our inverse design approach and utilizing a more accurate physical modeling method EMNN, the modeling errors can be well settled even with a large-scale and nonlinear computing architecture.

**Figure 4:**
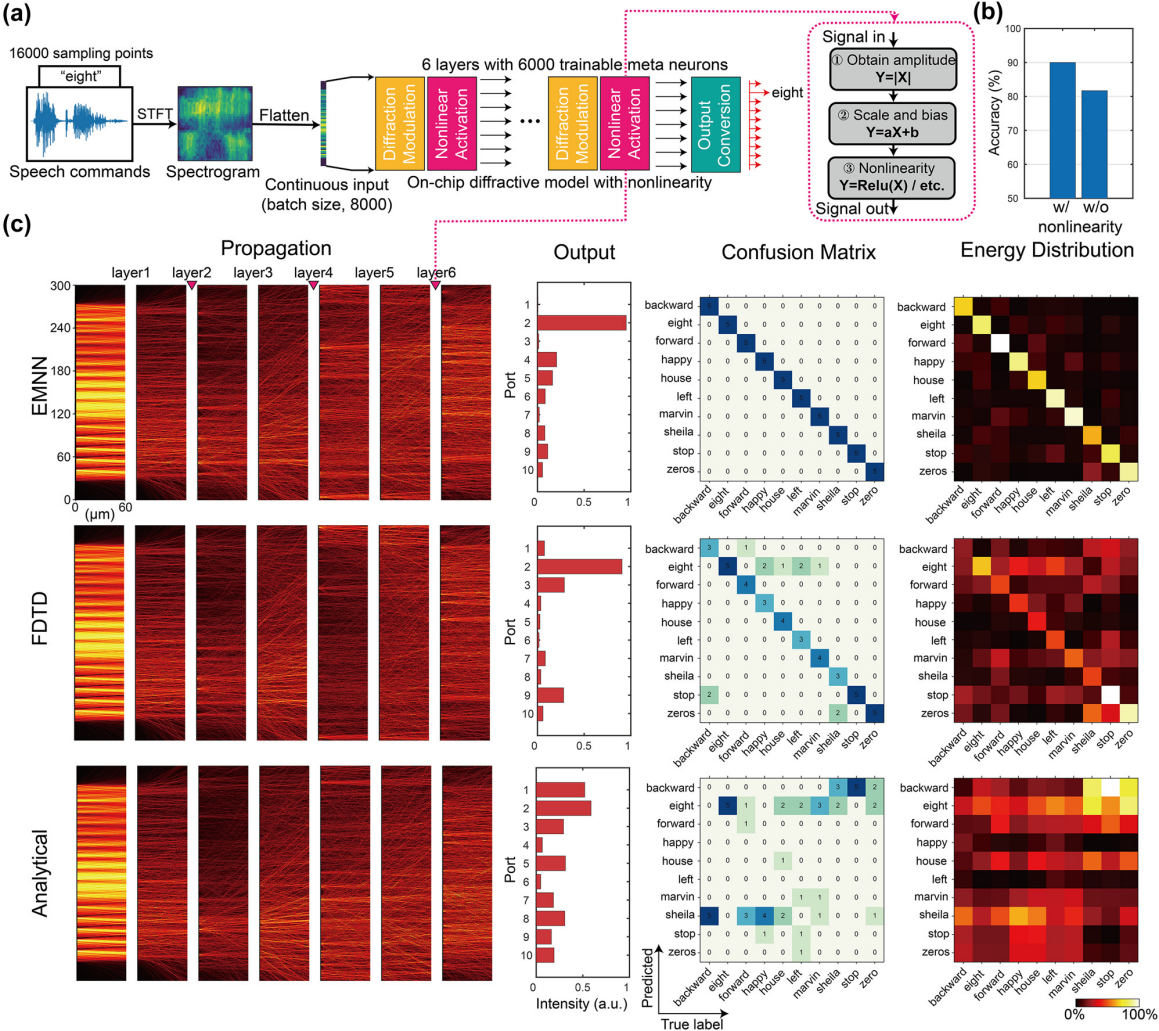
Six-layer nonlinear ONN design on the task of speech command recognition. (a) The schematic of ONN architecture composed of preprocessing, diffraction modulation, nonlinear activation, and output conversion. (b) Performance comparison between linear ONN and nonlinear ONN on speech command classification. (c) Comparison results using different forward inference methods (EMNN, FDTD, and ASM) on the same physical structure. Here, the propagation and outputs of FDTD serves as the ground truth. The first column shows the forward propagation profile with the speech “eight” inputted. The second column shows the output energy distributions. The third column shows the confusion matrices. The last column shows the energy distribution matrices.

Therefore, a more complex on-chip optical diffraction computation architecture is designed, as sketched in Figure 4a. The network is expanded to six layers, with the introduction of three nonlinear layers within the network. It should be noticed that the nonlinearity (Figure 4a) is trainable to increase the learning capability of the ONN and also test the efficacy of EMNN under a more complicated circumstance (see Figure S7). This nonlinearity can be potentially realized by electronic photodetectors. With nonlinearity introduced, the learning ability of the network can be strengthened and produce higher accuracy in classification (see Figure 4b).

Previous research has explored the use of optical computing for audio classification tasks, such as vowel classification [10]. However, these tasks involve a small number of categories and low-dimensional features so as to be compatible with the totally linear optical computing framework. In this study, we choose the Speech Commands dataset. This dataset comprises a total of 34 categories of speech data, from which 10 categories are selected for our classification task. The dataset consists of 20,000 samples in total, where 16,000 samples serve as training set and 4,000 samples serve as testing set. Each sample is typically a speech clip of approximately 1-second-long duration, with a sampling rate of 16,000 Hz. These speech samples are recorded and curated from different speakers using various recording devices, and thus accents, background noise, and device-specific artifacts bring great challenges to the classification task. For such a complex task, a simple shallow linear optical network is inadequate. Thus, a deep nonlinear optical network is required to address this problem. Meanwhile, EMNN method is leveraged to increase the accuracy of physical modeling and enhance the efficiency and fidelity of the design. The details of training can be found in Supplementary Text 3.

The ten classification categories are “backward,” “eight,” “forward,” “happy,” “house,” “left,” “marvin,” “Sheila,” “stop,” and “zero.” The testing results using our proposed inverse design method are shown in the first two columns of Figure 4c. A randomly selected test sample, whose true label is “eight,” is used for forward inference. The first column displays the wavefront propagation profiles computed by different forward solvers, along with their corresponding output results. It can be observed that for the sample, all three solvers correctly classified it into the second class, “eight.” However, the ten output results of the ASM solver significantly deviated from the true output signal intensity values obtained from FDTD simulation.

Limited by computational resources, 5 samples are randomly selected from each class to form a test set with 50 samples. In the last two columns of Figure 4c, there are the testing results using different forward solvers on the test set. It can be observed that the EMNN method achieved sufficient accuracy in physical modeling. The classification accuracy, validated using the FDTD solver, can reach 80 %, even though the network’s depth and complexity are increased. While the analytical method achieves only 16 % in classification accuracy, marginally better than random guessing.

## 3 Conclusions

Here, we present reliable, efficient, and flexible inverse design of large-scale and high-DOF intelligent computing metasystems by incorporating physics-inspired design into deep learning. This physics-inspired method is named EMNN and consists of EMNN Netlet and Huygens–Fresnel Stitch. EMNN Netlet is capable of predicting the EM field distribution accurately based on input field of arbitrary variation, while Huygens–Fresnel Stitch method combines these local EM field distributions to obtain an arbitrarily large EM field distribution. EMNN helps guarantee both high efficiency and high fidelity of the design and has great scalability to cope with devices with large-scale, high DOF, and complex functionality. With its help, we successfully perform inverse design of on-chip diffraction neural networks to accomplish complex tasks such as handwritten digit recognition and speech command classification.

### 3.1 Reliable and efficient inverse design

EMNN is applied to realize reliable and efficient inverse design by providing a much more accurate and rapid tool of EM field prediction. On one hand, analytical models, due to their simplification and abstraction of actual physical processes, can quickly compute an approximate result. However, the accuracy is not high, and through multiple layers of structure, errors accumulate, resulting in a loss of fidelity in the final device performance. On the other hand, numerical methods solve time-dependent partial differential equation systems using difference methods, providing accurate results, but at the cost of significant spatial and temporal computing resources. Utilizing data-driven deep learning approaches to learn the diffraction process can eliminate the need for time-consuming and resource-intensive numerical simulation processes, enabling direct accurate predictions of EM field. Furthermore, the end-to-end learning architecture does not necessitate specialized physical knowledge or analytical capabilities. The proposed EMNN method improves the accuracy by two orders of magnitude compared to ASM (see Figure 3c and d) and ensures consistent high precision in both near-field and far-field scenarios (see Figure 3c). In addition, its output is differentiable with respect to the input, thus allowing for gradient-descent training of the system. Although EMNN is slightly slower than ASM, it is 17,000 times faster than numerical simulations (see Table S1). EMNN, an accurate and fast forward solver, ensures high fidelity and efficiency of the design.

### 3.2 Scalable and flexible network architecture

Our paradigm exhibits enhanced flexibility compared to previous deep learning-based inverse design methods. Our forward evaluation network incorporates the variations of the input source. In addition, the stitching strategy of Huygens–Fresnel Stitch enables strong scalability of network structure. In previous approaches, the lack of scalability in network structure restricts the algorithm to adjusting parameters within fixed-size regions. However, with the introduction of Huygens–Fresnel Stitch, the network exhibits strong scalability. Taking the inverse design of optical neural networks as an example, EMNN can be used to design ONN of any width and depth. More importantly, previous deep learning frameworks predict indirect physical quantities such as transmittance or spectrum, tailored to the specific task requirements. In contrast, EMNN can directly predict the EM field components. If the transmittance is desired, it can be calculated using the EM field components. The scalability and flexibility of EMNN meets unprecedented inverse design requirements for arbitrary scale, high DOF, and complex functionalities beyond the capability of other deep-learning-based methods (see Table S2).

### 3.3 Physics-inspired design

EMNN fuses physics-inspired design with deep learning technology to achieve better performance. Firstly, considering that the EM field itself is a complex field with phase and amplitude, we encode both the EM field information and diffraction modulation information into complex tensors. Furthermore, considering that light propagation can be viewed as a linear process of Fourier filtering, we introduce a network structure with complex-valued linear connections in EMNN Netlet. The physics-inspired design aids EMNN in extracting the underlying physical information hidden within the data, enabling the network to inherently meet the physical constraints of the system (see Figure S4). This grants EMNN a generalization capability that surpasses traditional network architectures, ensuring high-precision predictive capabilities. For Huygens–Fresnel Stitch, this method is inspired by the Huygens–Fresnel principle, the fundamental principle of optical field propagation. Leveraging this physics-inspired approach, we achieve scalability of the network and have advantages in accuracy over other nonphysics-inspired stitching strategies. Lastly, the physics-inspired method possesses strong interpretability.

### 3.4 Limitation and future work

The work shows great potential for further discussion and expansion. We have demonstrated the design of a metasystem composed of 1D metamaterials, and if we extend 1D tensors in EMNN to 2D, we can achieve efficient and accurate inverse design of 2D metamaterials. Additionally, by increasing the number of channels in the tensor, where each channel represents a different frequency component of the EM field, we can perform inverse design of devices based on frequency-domain responses. The element-wise multiplication operation in the network structure corresponds to the physical process of diffraction modulation, and it can be replaced by other mathematical operations depending on the specific physical process.

In terms of on-chip optical diffractive metasystems, although modeling errors are minimized, the device performance remains to be explored as the scale increases and the number of layers grows further. This calls for a brand-new analog computing architecture that is more robust against modeling errors. In terms of the network architecture, the training cost remains high, and there is a desire to incorporate more physical priors or physics-inspired approaches to realize semi-supervised or unsupervised paradigms. Our work is a demonstration of the integration of data-driven deep learning methods with sciences and engineering. In recent years, AI has been extensively applied in scientific and engineering domains, such as protein structure prediction [54], [55], [56], weather forecasting [57], [58], etc. More generally, it is anticipated that inspiration from principles in electromagnetics, mechanics, thermodynamics, or other disciplines help construct novel, more powerful, and more interpretable machine learning paradigms.

## Supplementary Material

Supplementary Material Details
