# Supplementary material for: Reliable, efficient, and scalable photonic inverse design empowered by physics-inspired deep learning
Source: Nanophotonics. 2025 Jan 27;14(16):2799–810. doi: 10.1515/nanoph-2024-0504 (PMC12338875; doi:10.1515/nanoph-2024-0504)
Supplement: Supplementary file 1 — Supplementary Material Details [file j_nanoph-2024-0504_suppl_001.docx]

***Supplementary Material***

Reliable, Efficient and Scalable Photonic Inverse Design Empowered by Physics-inspired Deep Learning

*Guocheng Shao^2,3^*^†^*, Tiankuang Zhou^1,4^*^†^*, Tao Yan^3^, Yanchen Guo^2,3^, Yun Zhao^2,3^,*

*Ruqi Huang^2^* and Lu Fang^1,4,5^**

^1^Department of Electronic Engineering, Tsinghua University, Beijing 100084, China.

^2^ Shenzhen International Graduate School, Tsinghua University, Shenzhen 518071, China.

^3^ Department of Automation, Tsinghua University, Beijing 100084, China.

^4^ Beijing National Research Center for Information Science and Technology, Tsinghua University, Beijing 100084, China.

^5^ Institute for Brain and Cognitive Sciences, Tsinghua University, Beijing 100084, China.

E-mail: [ruqihuang@sz.tsinghua.edu.cn](mailto:ruqihuang@sz.tsinghua.edu.cn); [fanglu@tsinghua.edu.cn](mailto:fanglu@tsinghua.edu.cn)

**Table S1.** Forward evaluation time of different methods and device sizes

| **Device size** | 150 um | 300 um | 600 um |
| --- | --- | --- | --- |
| **EMNN** | 0.89s | 1.71s | 3.67s |
| **Simulation (FDTD)** | 3h19min | 6h27min | 13h3min |
| **Analytical (ASM)** | 0.94s | 0.98s | 1.23s |

**Table S2.** Comparisons of EMNN and other deep-learning-based inverse design methods in terms of the device size, design DOF and device functionality.

| **No.** | **Device Size** | **Device DOF** | **Input Field DOF** | **Output Field DOF** | **Device Functionality** |
| --- | --- | --- | --- | --- | --- |
| Proposed Method | ~300um  (arbitrarily large) | 6000 float(arbitrarily large) | 10000 complex(arbitrarily large) | 10000 complex(arbitrarily large) | intelligent computing metasystems |
| Ref [1] | ~0.5um | 8 float | 0 | 200 float | light scattering nanoparticles |
| Ref [2] | ~3um | 20 float | 0 | 200 float | transmission spectrum of multilayer structures |
| Ref [3] | ~2um | 64*64 binary | 1 binary（polarization） | 31*3 float | desired reflection spectrum of metasurfaces |
| Ref [4] | ~0.7um | 4 float | 0 | 3 float | silicon nanostructure generating color |
| Ref [5] | ~20um | 64*64 binary | 0 | 176 complex | transmittance/reflectance of meta-atom(polarization manipulation) |
| Ref [6] | ~10mm | 8*8 binary | 0 | 1 float | 2D focusing and abnormal reflection metasurfaces |
| Ref [7] | ~1um | 6 float | 0 | 100 float | reflectance spectrum of plasmonic stack metamaterial |
| Ref [8] | arbitrarily large | arbitrarily large | 0 | arbitrarily large | near-field manipulation of metasurfaces |

**Table S3**. Performances of the simulation methods(corresponding to Figure S8).

| Method | Loss (in specific case) | Wallclock time (in specific case) | Algorithm complexity | Computing platform |
| --- | --- | --- | --- | --- |
| ASM | 0.7799 | 0.7005s | *O(n)* | GPU Ref [9] |
| EMNN (Ours) | **0.2138** | **0.3489s** | *O(n)* | GPU |
| 2D-FDTD | 0.5309 | 3.50s | *>O(n^2^)* | CPU Ref [10] |
| FDTD | Ground truth | 31.87s | *>O(n^3^)* Ref [11] | CPU |

**Supplementary Text**

1. **Device Design Configurations**

On-chip optical neural network is fabricated with Silicon-on-insulator (SOI), and each layer of metastructures (metalines/metagratings) on the chip is designed to modulate the wavefront of the passing light, and multiple layers of metastructures are cascaded together to form an on-chip optical neural network with super computational power. Each layer of the metastructure consists of a number of silica slots with different lengths; each slot has a fixed thickness and width, as illustrated in Figure S2b. Merely by changing its length, the optical field can be modulated in phase or amplitude. Thus, each slot can be considered as a learning parameter of the on-chip diffractive optical network. In our work, we fix the width of the slots to be 150 nm, the alignment period to be 300 nm, the thickness to be 220 nm in line with that of the silicon layer, and the length can be chosen continuously in the interval of 0-900 nm. Alternatively, if considering the manufacturability, a number of discrete values can be chosen in this interval, e.g., 0, 300 nm, 600 nm, 900 nm, which is sufficient for the slots to produce a -pi-to-pi phase or a 0.5-to-1 amplitude modulation.

The distance between adjacent metastructures is set to a fixed value (60um in this work) or as a learning parameter that can be trained in inverse design, depending on the task. The operating wavelength of the device is 1550 nm.

1. **Data Description**

The input wavefront is 900 nm wide in the z-direction, and the waveform is spatially sampled with a period of 30 nm. An example of the input wavefront in y-z plane is displayed in Figure S2a. Considering the EM field propagates in a fixed mode on the chip, we can thus represent it in the form of the magnetic field component *Hy* at the center of the chip. As a result, the input wavefront can be represented in the form of a complex-valued vector.

The given input structure, an array of 16 silica slots of varying lengths, is 4.8 um wide in the z-direction, with a spatial sampling period of 300 nm (which can be set smaller if manufacturability is omitted and thereby the DOF of device design is higher), which is up-sampled by a factor of 10 in order to keep the spatial sampling period the same in the data processing process. An example of the given structure is shown in Figure S2b. Accordingly, the input structure can be represented as a vector of real numbers.

Similar to the input wavefront, the output wavefront presented in Figure S2d is also represented by the magnetic field component *Hy* at the center of the chip, but is wider than the input wavefront in the z-direction, scaling up to 4.8 um, due to the diffractive property that the field expands with propagation.

1. **Training of diffractive ONN**

The training of our on-chip diffractive chip follows the same rule as those in [12, 13].

Diffractive ONNs utilize the principles of optical diffraction and wave propagation to implement deep learning functions in a fully optical manner. During forward propagation, light passing through each neuron in a given layer of metaline is treated as a secondary wave source. The output amplitude and phase of the wave are determined by the output of EMNN fed with the input wavefront and the trainable metaline structure, which encodes trainable parameters such as phase and amplitude (This part used to use ASM or ERI to give an approximate forward evaluation). This propagation follows electromagnetic principles, such as Huygens-Fresnel, the physical constraints of the optical system.

Backward propagation involves computing gradients of a loss function—typically based on the discrepancy between the target and output light intensity distributions—through successive layers of metalines. Techniques like stochastic gradient descent or Adam optimization are employed to iteratively update the transmission coefficients. To address practical limitations, these coefficients are pre-trained in simulations and then mapped to physical structures like metalines.

**Supplementary Figures**


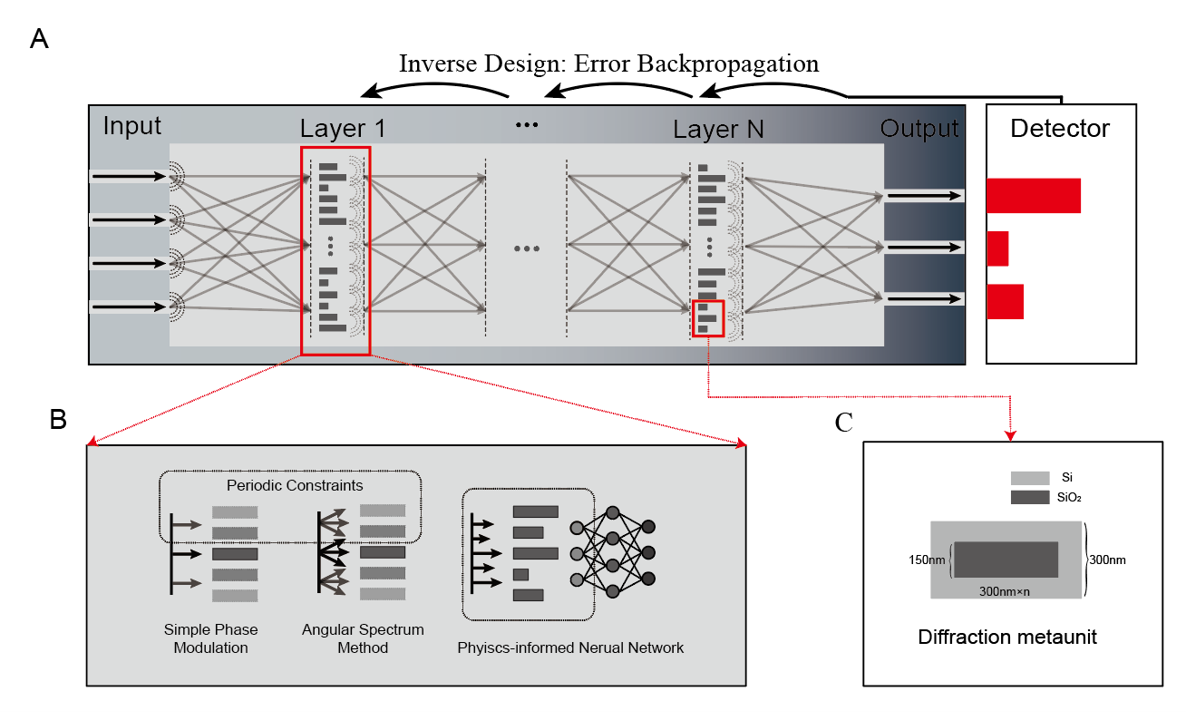


**Figure S1 On-chip diffractive computing metasystem.**

**(A)** Schematic of the diffractive computing metasystem. **(B)** Illustration of analytical and deep-learning-based forward problem solvers. **(C)** Schematic of the diffractive meta atom.


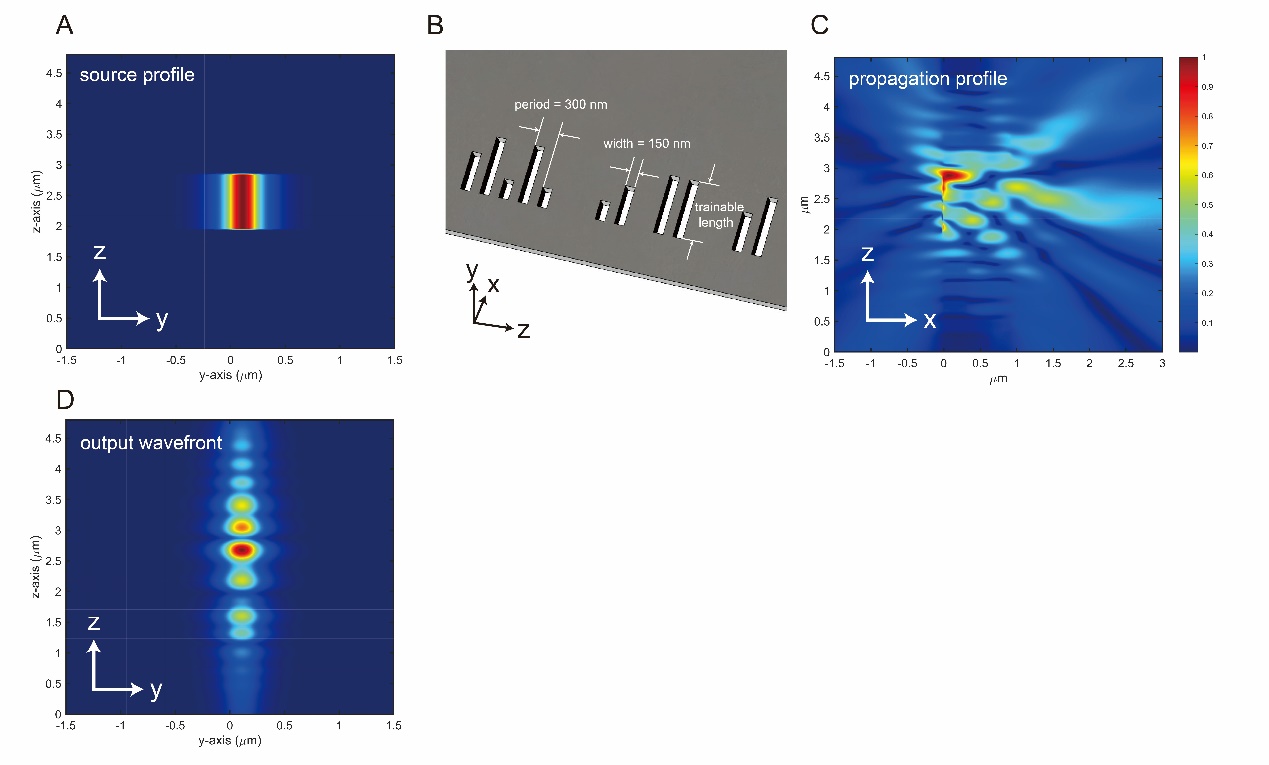


**Figure S2 Slab-mode light propagation.**

**(A)** The y-z plane profile of the input wavefront, which is given in advance. **(B)** Conceptional view of the diffractive metaline. **(C)** Slab-mode propagation profile (in the x-z plane) simulated by FDTD. **(D)** The y-z plane profile of the output wavefront.


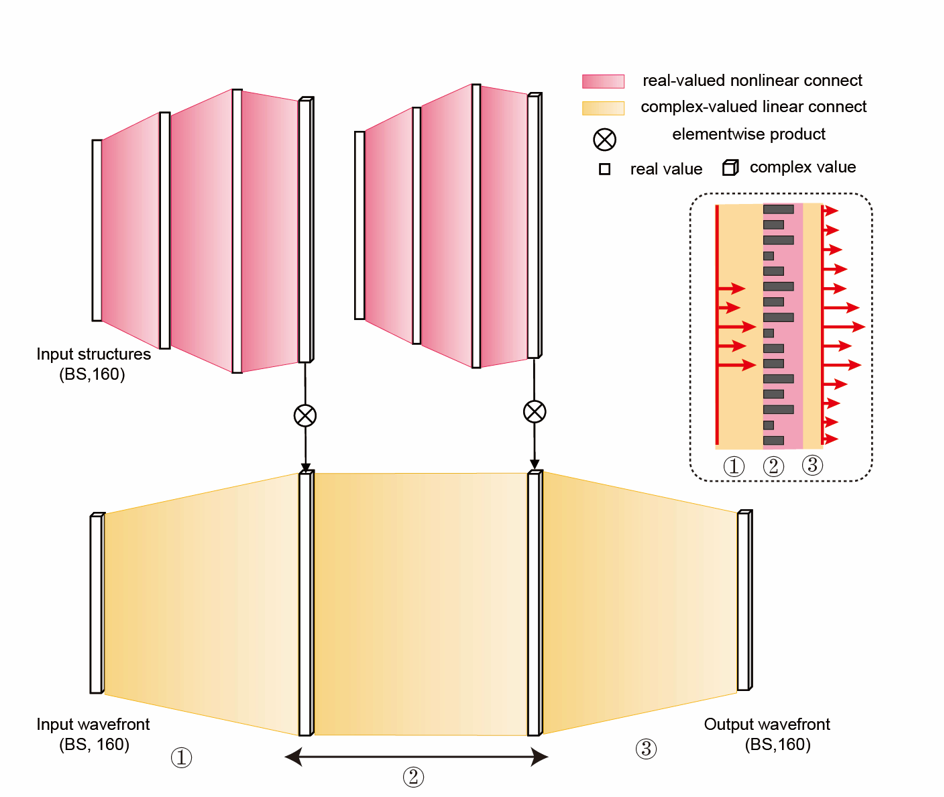


**Figure S3 A detailed architecture illustration of interpretable EMNN Netlet.**

The network is physically interpretable based on its correspondence to real physical structures. The propagation process of the input wavefront can be divided into three stages: 1) free-space propagation before reaching the silica slots (the distance is short but exists); 2) diffractive modulation process in this region of the silica slots; 3) another free-space propagation process after leaving the silica slots.


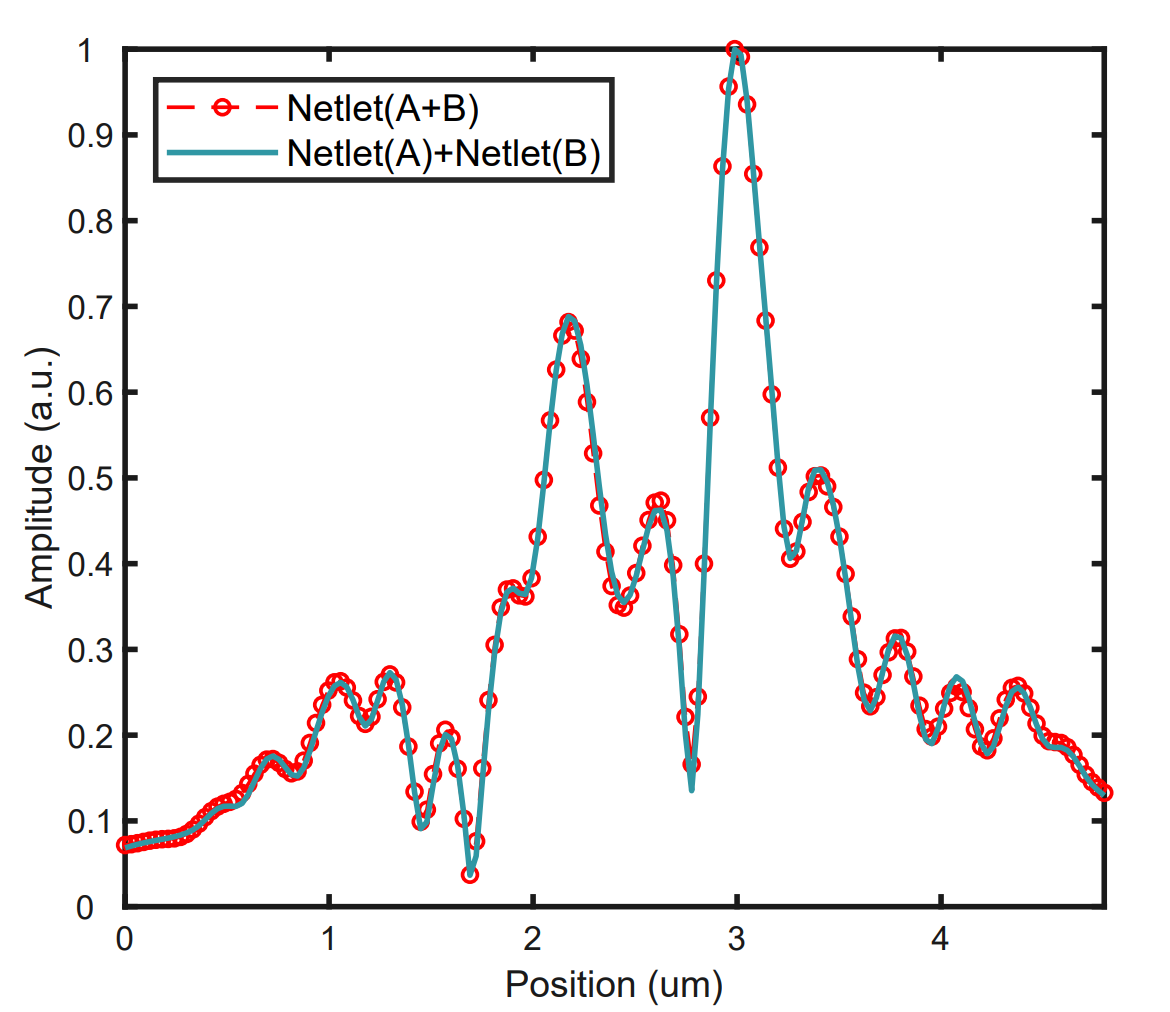


**Figure S4** **Linearity of EMNN Netlet.**

The waveform comparison of the output produced by superposed inputs and the superposed outputs, verifying the linearity of EMNN Netlet and aligning with the physical system.

**
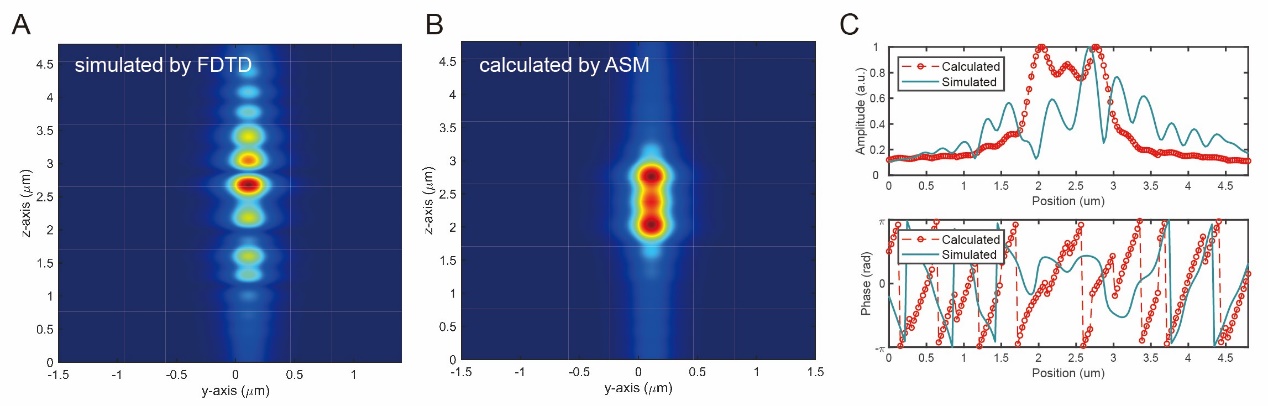
**

**Figure S5** **Performance comparison of proposed and analytical approaches.**

**(A)** The y-z plane profile of the output wavefront simulated by FDTD. **(B)** The y-z plane profile of the output wavefront calculated by the analytical model. **(C)** The amplitude and phase comparisons of simulated and calculated wavefront.


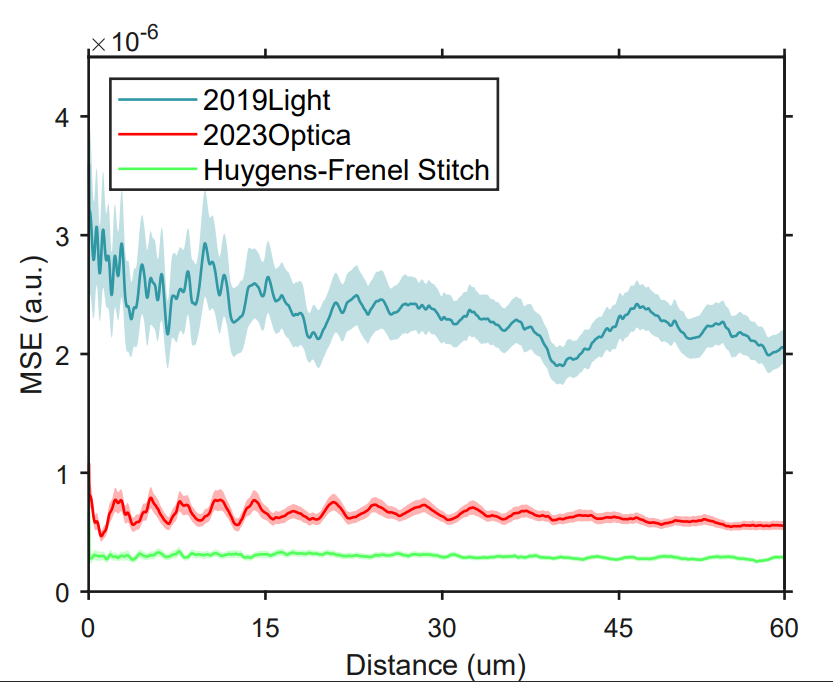


**Figure S6** **Performance comparison of different stitching strategies.**

Mean square errors versus propagation distance using three different stitching strategy.

**
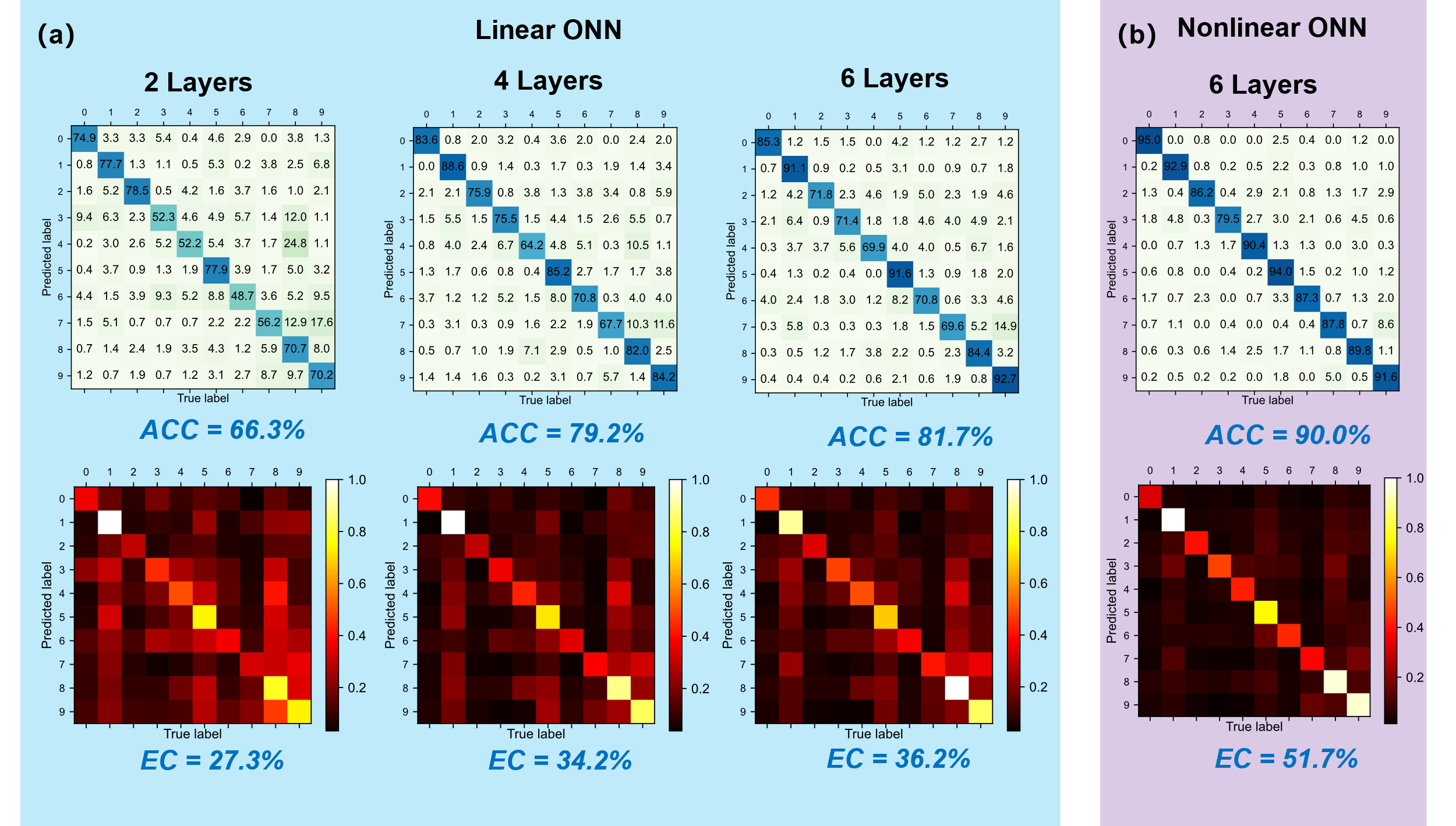
**

**Figure S7** **Performance of ONN with different setups testing on Speech Command dataset.**

**(a)** Confusion and energy matrices of linear ONN with 2, 4 and 6 layers of metalines. **(b)** Confusion and energy matrices of nonlinear ONN with 6 layers of metalines. (ACC, accuracy, EC, energy concentration)


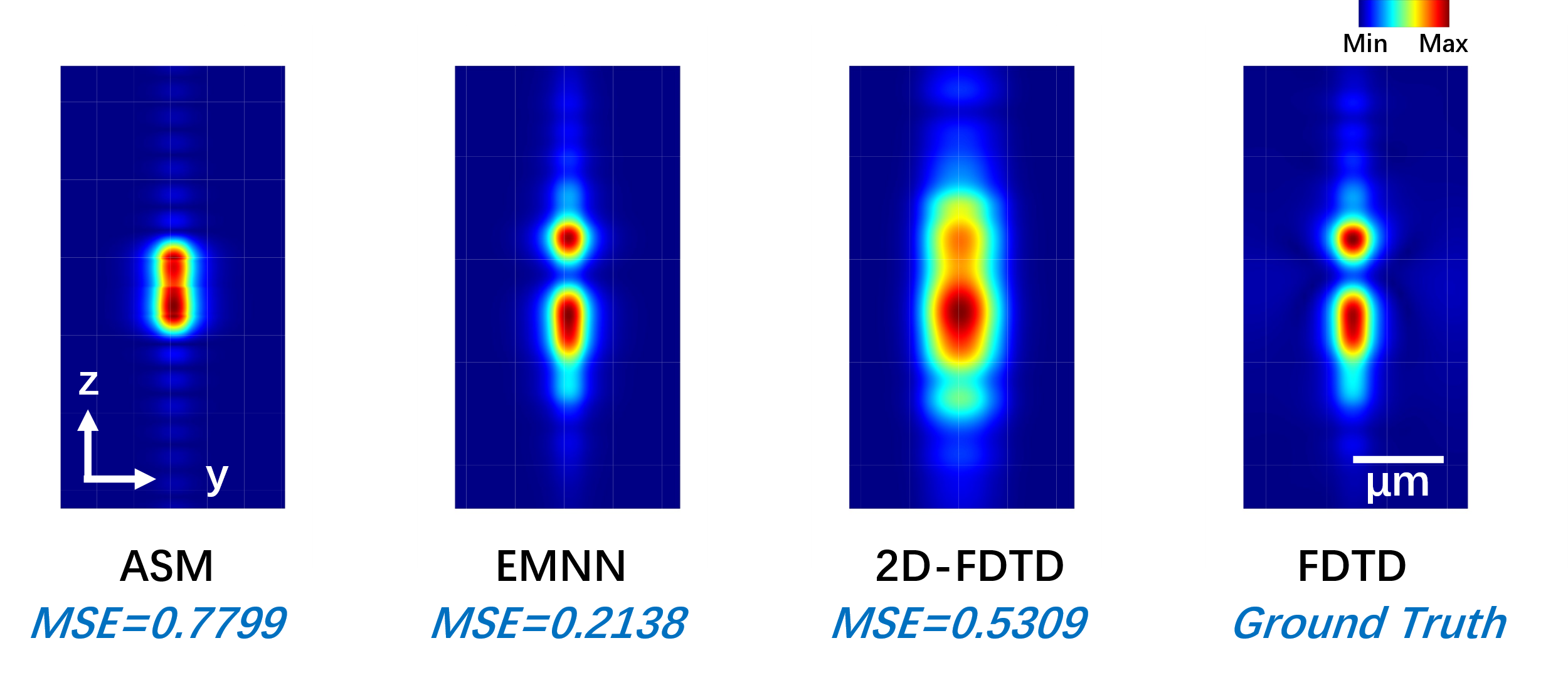


**Figure S8** Simulated intensity profiles of the mode source modulated by metalines using ASM, EMNN, 2D-FDTD and FDTD respectively. The maximum of the light intensity is normalized to 1(a.u.). The MSEs marked in blue indicate the simulation accuracy (a smaller loss indicates a higher simulation accuracy) compared with the ground truth, FDTD.


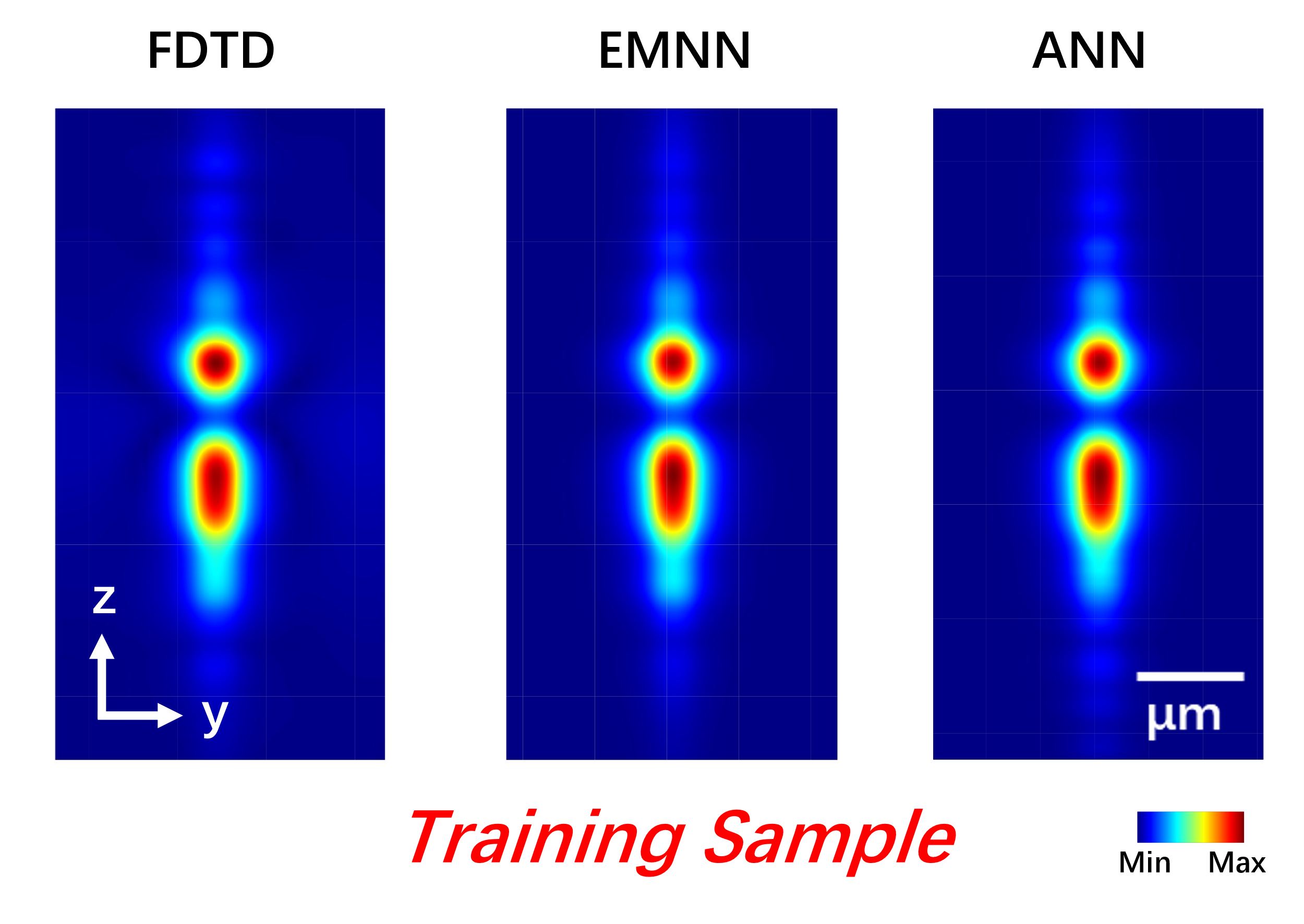


**Figure S9** Profiles of results of a training sample (the input source is a plane wave) simulated by FDTD, EMNN and ANN respectively.


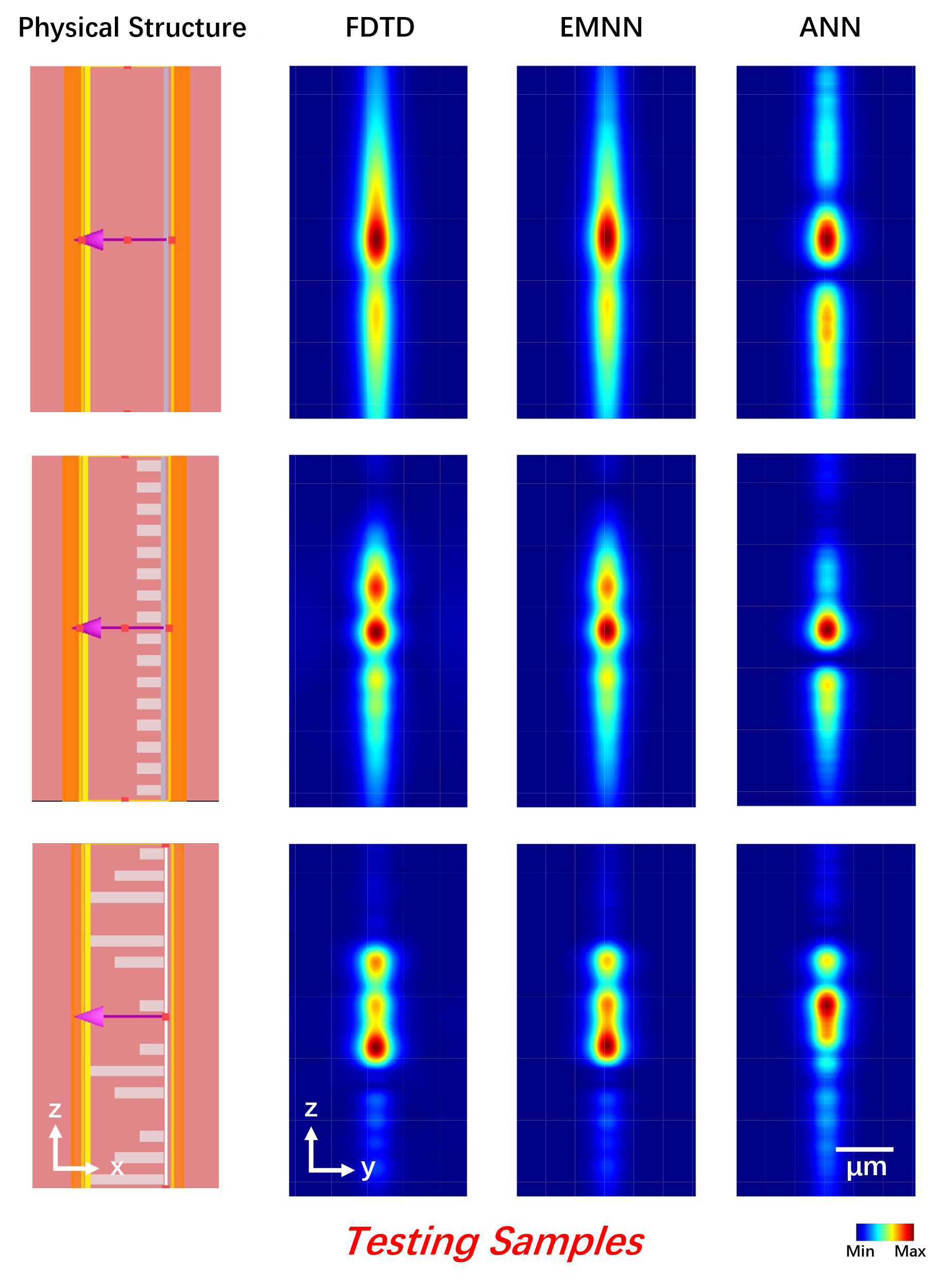


**Figure S10** Physical structures and corresponding profiles of results of testing samples (the input source is not a plane wave) simulated by FDTD, EMNN and ANN respectively.

**References**

[1] J. Peurifoy et al., “Nanophotonic particle simulation and inverse design using artificial neural networks,” SCIENCE ADVANCES, 2018.

[2] D. Liu, Y. Tan, E. Khoram, and Z. Yu, “Training Deep Neural Networks for the Inverse Design of Nanophotonic Structures,” ACS Photonics, vol. 5, no. 4, pp. 1365–1369, Apr. 2018, doi: 10.1021/acsphotonics.7b01377.

[3] W. Ma, F. Cheng, Y. Xu, Q. Wen, and Y. Liu, “Probabilistic Representation and Inverse Design of Metamaterials Based on a Deep Generative Model with Semi‐Supervised Learning Strategy,” Advanced Materials, vol. 31, no. 35, p. 1901111, Aug. 2019, doi: 10.1002/adma.201901111.

[4] L. Gao, X. Li, D. Liu, L. Wang, and Z. Yu, “A Bidirectional Deep Neural Network for Accurate Silicon Color Design,” Advanced Materials, vol. 31, no. 51, p. 1905467, Dec. 2019, doi: 10.1002/adma.201905467.

[5] Z. Liu, D. Zhu, K. Lee, A. S. Kim, L. Raju, and W. Cai, “Compounding Meta‐Atoms into Metamolecules with Hybrid Artificial Intelligence Techniques,” Advanced Materials, vol. 32, no. 6, p. 1904790, Feb. 2020, doi: 10.1002/adma.201904790.

[6] R. Zhu et al., “Phase-to-pattern inverse design paradigm for fast realization of functional metasurfaces via transfer learning,” Nat Commun, vol. 12, no. 1, p. 2974, May 2021, doi: 10.1038/s41467-021-23087-y.

[7] J. Xiong et al., “Real‐Time On‐Demand Design of Circuit‐Analog Plasmonic Stack Metamaterials by Divide‐and‐Conquer Deep Learning,” Laser &amp; Photonics Reviews, vol. 17, no. 3, p. 2100738, Mar. 2023, doi: 10.1002/lpor.202100738.

[8] T. B. Kanmaz, E. Ozturk, H. V. Demir, and C. Gunduz-Demir, “Deep-learning-enabled electromagnetic near-field prediction and inverse design of metasurfaces,” Optica, vol. 10, no. 10, p. 1373, Oct. 2023, doi: 10.1364/OPTICA.498211.

[9] Nvidia GeForce GTX 1080 Ti.

[10] Intel(R) Xeon(R) CPU E5-2620 v4 @ 2.10GHz 2.10 GHz (2 processors).

[11] Phan, T. et al. High-efficiency, large-area, topology-optimized metasurfaces. Light Sci Appl 8, 48 (2019).

[12] X. Lin et al., “All-optical machine learning using diffractive deep neural networks,” Science, vol. 361, no. 6406, pp. 1004–1008, Sep. 2018, doi: [10.1126/science.aat8084](https://doi.org/10.1126/science.aat8084).

[13] T. Zhou et al., “Large-scale neuromorphic optoelectronic computing with a reconfigurable diffractive processing unit,” Nat. Photonics, vol. 15, no. 5, pp. 367–373, May 2021, doi: [10.1038/s41566-021-00796-w](https://doi.org/10.1038/s41566-021-00796-w).
